# Efficiency of Biobran/MGN-3, an Arabinoxylan Rice Bran, in Attenuating Diabetes-Induced Cognitive Impairment of the Hippocampus via Oxidative Stress and IR/Akt/NF-*κ*B in Rats

**DOI:** 10.1155/2023/8248576

**Published:** 2023-07-19

**Authors:** Heba M. Abdou, Fatma A. Hamaad, Ghada M. Abd Elmageed, Mamdooh H. Ghoneum

**Affiliations:** ^1^Department of Zoology, Faculty of Science, Alexandria University, Alexandria, Egypt; ^2^Department of Biochemistry, Faculty of Science, Alexandria University, Alexandria, Egypt; ^3^Department of Surgery, Charles R. Drew University of Medicine and Science, Los Angeles, CA 90059, USA; ^4^Department of Surgery, University of California Los Angeles, Los Angeles, CA 90095, USA

## Abstract

Type 2 diabetes mellitus (T2DM) is a common metabolic disease accompanied by cognitive impairment, hippocampal malfunctioning, and inflammation. Biobran/MGN-3, an arabinoxylan rice bran, has been shown to have an antidiabetic effect in streptozotocin-induced diabetic rats. The present study investigates Biobran's effect against diabetes-induced cognitive impairment and synaptotoxicity in the hippocampus via oxidative stress and the IR/A/NF-*κ*B signaling pathway in rats. Diabetes was induced via i.p. injection of streptozotocin (STZ) (40 mg/kg BW); STZ-treated rats were then administered Biobran (100 mg/kg BW) for 4 wks. Biobran supplementation improved motor coordination and muscular strength, as assessed by Kondziella's inverted screen test. Biobran also improved concentration levels of glutathione (GSH), antioxidant enzymes, acetylcholine (ACh), dopamine, serotonin, insulin receptor (IR), and alpha serine-threonine protein kinase (Akt); it protected against elevated levels of glucose, total cholesterol, triglycerides, oxidative stress markers, TBARS, NO, AChE, and MAO; and it significantly decreased inflammatory cytokines levels of IL-1*β*, NF-*κ*B, TNF-*α*, and amyloid *β*_1-42_. Moreover, Biobran ameliorated hippocampal histological alterations. Immunohistochemical observations showed that Biobran reduced overexpression of hippocampal synaptophysin and Ki67 relative to untreated diabetic rats. Biobran may ameliorate hippocampal alterations in diabetic rats via its antidiabetic, antiproliferative, anti-inflammatory, antiapoptotic, and antioxidant effects.

## 1. Introduction

Diabetes mellitus (DM) is one of the most common chronic endocrine metabolic disorders. It is characterized by hyperglycemia [1] and its most common form, type 2 diabetes mellitus (T2DM), is characterized by relative insulin insufficiency and insulin resistance [2]. T2DM is associated with long-term complications affecting the brain, heart, blood vessels, kidneys, and eyes [3]. Moreover, chronic hyperglycemia through the polyol pathway and the oxidative stress state induced by the increased formation of reactive oxygen species have been proposed as major pathophysiological links between T2DM progression and the onset of diabetic complications [4]. Similarly, the pathogenesis of diabetes and diabetic complications is associated with increased inflammation [5]. Diabetes and insulin-related effects are associated with changes in serotonin (5-HT) and dopamine (DA) neurotransmitters, and the insulin signaling mechanism alpha serine-threonine protein kinase (Akt) has been implicated in hippocampal neurogenesis [5]. Akt's signal transduction pathway is comprised of Akt, the Bcl-xL/Bcl-2-associated death promoter (Bad), and nuclear factor kappa B (NF-*κ*B), and this pathway is involved in cell metabolism, differentiation, proliferation, and apoptosis [6].

Recently, evidence has grown indicating that chronic T2DM patients can have neurobehavioral changes and various intracranial neuropathies [7]. Several reports have found a strong relationship between DM and cognitive deficits, neuronal impairment, and dementia [7, 8]. The primary clinical features associated with cognitive impairment induced by diabetes have included psychomotor retardation, learning and memory impairment, and decreased mental flexibility [8]. The hippocampus is particularly sensitive to DM's chronic hyperglycemia which can lead to cognitive impairment, to delayed responses to surroundings, and to hippocampal neuron number reduction and abnormal morphology [6].

Many antidiabetic drugs are being explored in clinical practice, but unfortunately those that are available are impotent against DM complications, including cognitive impairment, and they are associated with disagreeable side effects [8]. Given this situation, it is immensely important to investigate novel and multitargeted natural products that can mitigate DM pathogenesis. Biobran/MGN-3, a rice bran arabinoxylan, is a polysaccharide containing beta-1,4 xylopyronase hemicellulose [9] that has been proven to be an effective antioxidant that prevents free radical formation and enhances the antioxidant defense system [10]. We have also recently reported Biobran's beneficial effects on aging [11, 12] and neurodegenerative diseases [10], and recent studies have furthermore reported the antidiabetic effect of Biobran in streptozotocin-induced diabetic rats [13]. The current study investigates Biobran's effect against diabetes-induced cognitive impairment and hippocampal synaptotoxicity via oxidative stress and the IR/Akt/NF-*κ*B signaling pathway in rats. We hypothesized that Biobran's distinct antioxidant properties would relieve diabetes-induced cognitive impairment. This hypothesis was tested by systematically investigating Biobran's therapeutic effects against diabetes-induced cognitive impairment and hippocampal synaptotoxicity via oxidative stress and the IR/Akt/NF-*κ*B pathway in rats.

## 2. Materials and Methods

### 2.1. Biobran

Biobran is a processed hemicellulose that is obtained by reacting rice bran hemicelluloses with multiple carbohydrate hydrolyzing enzymes from shiitake mushrooms. The main chemical structure of Biobran is an arabinoxylan, with a xylose in its main chain and arabinose in its side chain [9]. Biobran is water soluble and solutions of Biobran were freshly prepared every day by mixing Biobran with saline (0.9% w/v). The dose used in the present study (100 mg/kg body weight every day for 4 wks) was chosen in accordance with our previous work [14]. 

### 2.2. Chemicals

Streptozotocin (STZ, 98% purity) was obtained from Sigma-Aldrich Chemical Company (St. Louis, MO, USA), as were total cholesterol (TC) and triglyceride (TG). Insulin was obtained from Linco Research, USA. Nitric oxide (NO) was obtained from Bio Systems S.A. Glucose, thiobarbituric acid reactive substances (TBARS), reduced glutathione (GSH), superoxide dismutase (SOD), and glutathione peroxidase (GPx) were obtained from Biodiagnostic Co. (Cairo, Egypt). Synaptophysin and Ki67 antibodies were obtained from Abcam Co. (Cambridge, UK). Other reagents were purchased from MyBioSource Co. (San Diego, USA). Daiwa Pharmaceutical Co. Ltd. (Tokyo, Japan) kindly provided Biobran (99% purity).

### 2.3. Diabetic Model

The diabetic model in this experiment used STZ and a high-fat diet (HFD) consisting of 66.5% commercial feed (Labina), 13.5% cholesterol, and 20% sugar. HFD feeding was used to induce insulin resistance and STZ at low dose was used to cause initial dysfunction in beta cells, which closely mimics the natural metabolic events of human T2DM.

### 2.4. Animals and Treatment

24 male Wistar albino rats were obtained from the Animal Care Unit, Experimental Animal Center, Medical Research Institute, Alexandria University (Alexandria, Egypt). Rats weighed 170–180 g. Rats were separated into 4 groups of 6 and housed in a well-ventilated animal house in stainless steel wire cages. Rats were fed a basal diet, supplied with tap water ad libitum, and kept under constant environmental conditions at 22 ± 3°C temperature and a 12/12-h-light/dark cycle. The control group and Biobran group were fed commercial feed. The diabetic untreated group and diabetic plus Biobran group were fed a HFD. All animals were given their respective diets for two weeks, and on the 15th day, the animals in the diabetic groups were fasted for 12 h and intraperitoneally administered a single dose of STZ (40 mg/kg body weight) [15]. Control animals were given only vehicle (0.01 M citrate buffer, pH 4.5). Three days after STZ induction (on the 18th day of treatment), measurements of blood glucose were taken to confirm diabetes' establishment. Animals with postprandial glycaemia values ≥288 mg/dL were considered diabetic. Animals treated with Biobran were orally administered 100 mg Biobran per kg body weight every day for 4 wks [10]. No mortality was observed in any groups during the experiment. All animal handling and experimental procedures were performed according to the guidelines approved by Alexandria University Institutional Animal Care and Use Committee (ALEXU-IACUC), a member of the International Council for Laboratory Animal Science (ICLAS) (approval number: AU 04 22 06 27 3 01).

### 2.5. Behavioral Analysis

Kondziella's inverted screen test was used to measure animals' muscular strength in all limbs [16]. The inverted screen is made of wire 1 mm in diameter shaped into 12 mm squares over a total square mesh size of 43 cm. The screen border consists of wood beading 4 cm deep, preventing the rats from moving to the opposite side. Rats are placed in the screen center, following which the screen is rotated (declining the rat's head) for 120 seconds to a fully inverted position. Measurements were made of the time it took for the rat to fall off the screen.

### 2.6. Blood Sample Collection and Tissue Preparation

When the experiment finished, animals were given i.p. injections of a mixture of ketamine (100 mg/kg) and xylazine (5 mg/kg) to induce anesthesia, following which they were sacrificed by cervical dislocation. Blood samples from the abdominal aorta were drawn and collected into tubes. Serum was collected for measuring the concentration of glucose, insulin, triglycerides, and total cholesterol using commercial kits (Sigma-Aldrich Inc., St. Louis, MO, USA). The brain was harvested, and the hippocampus from one hemisphere was rapidly isolated on ice and stored at −80°C; the remaining hemisphere's hippocampus was immediately fixed in (10%) formalin for immunohistochemistry and histological analysis. 10% (w/v) tissue homogenates were prepared by combining 100 mg of hippocampus tissue with 1000 *μ*l of PBS buffer (pH 7.4) in Eppendorf tubes, centrifuging (2,000 × *g*, 10 min, 4°C), and then storing supernatants (−20°C) for later assays.

### 2.7. Biochemical Analysis

Serum levels of glucose were measured with a spectrophotometric assay kit. Serum levels of insulin were measured with sandwich ELISA kits (Linco Research, USA) according to the manufacturer's instructions. Serum levels of total cholesterol (TC) were assessed with a commercial kit (Sigma, USA). Serum concentrations of triglyceride (TG) were enzymatically measured with Sigma Chemical Co. kit.

Lipid peroxidation in the hippocampus was measured with thiobarbituric acid reactive substances (TBARS) following Ohkawa et al.'s method [17]. The level of nitric oxide (NO) was estimated calorimetrically by using commercial kits (Bio Systems S.A.). Reduced glutathione (GSH) was spectrophotometrically measured at 412 nm following Jollow et al.'s method [18]. Superoxide dismutase (SOD; EC: 1.15.1.1) and glutathione peroxidase (GPx; EC: 1.11.1.9) activities were ascertained in hippocampus tissues following Nishikim et al.'s [19] and Rotruck et al.'s [20] methods, respectively.

Acetylcholine (ACh), dopamine (DA), serotonin (5-HT), acetylcholinesterase (AChE; EC 3.1.1.7), and monoamine oxidase (MAO; EC 1.4.3.4) activity were estimated using ELISA kits (MyBioSource, San Diego, USA). Insulin receptor (IR), Akt, TNF-*α*, NF-*κ*B, IL-1*β*, and amyloid *β*_1-42_ were estimated by ELISA according to the manufacturer's instructions for the rat immunoassay kits (MyBioSource, San Diego, USA).

The glucose, insulin, TC, and TG assays required 10 *μ*l of serum. GSH, acetylcholine, and amyloid *β*_1-42_ assays required 50 *μ*l of homogenate. The MAO assay required 30 *μ*l of homogenate. The TBARS assay required 20 *μ*l of homogenate. The rest of the parameters required 10 *μ*l of homogenate.

### 2.8. Histological and Immunohistological Assessments

After fixation of hippocampal tissues in 10% buffered neutral formalin solution, the tissues were embedded in paraffin, sectioned, stained with hematoxylin and eosin (H&E), and examined microscopically for the evaluation of histopathological changes [21].

For immunohistochemical staining, xylene was used to deparaffinize paraffin sections (5 *μ*m thick) over 1-2 min. These were then rehydrated with ethanol of decreasing grades (100%, 95%, and 70% ethanol), using two changes each of 5 mins, with a final 5 min stage with distilled water. PBS was then used to rinse sections and 0.1% H_2_O_2_ was used to block for 30 min as an activity inhibitor of endogenous peroxidase. Following the PBS rinse, incubation of sections was conducted at room temperature (21°C) for 1 h in blocking solution (10% normal goat serum), followed by incubation (21°C) for 1 h with the primary antibody (synaptophysin and Ki67). PBS was again used to rinse sections, after which sections were incubated (21°C) with secondary biotinylated antibody for 20 min. PBS was used to rinse sections, and then enzyme conjugate “streptavidin-horseradish peroxidase” solution was applied over 10 min. Visualization of secondary antibody binding was carried out using 3,3′-diaminobenzoic acid (DAB) dissolved in PBS to which H_2_O_2_ was added for a concentration of 0.03% immediately before use. Finally, PBS was used to rinse sections and hemotoxylin (100 *μ*l, 2 drops) was used to counterstain the slides. Distilled water was used to wash the slides until sections became blue. Slides were dehydrated with increasing grades (70%, 95%, and 100%) of ethanol for 5 min each, cleared in xylene, and cover-slipped with histomount mounting solution.

Immunohistochemical assessments were carried out using five nonoverlapping fields (400x) of each section photographed randomly with an Olympus digital camera in the hippocampus. Every brain section and every marker were used to analyze total dentate gyral area.

### 2.9. Statistical Analysis

Results reported are mean ± standard error (SE); six rats were used per group (*n* = 6). The data were subjected to statistical analyses using a one-way analysis of variance (ANOVA) (PC-STAT, University of Georgia, 1985); groups were compared against each other using the LSD test. *p* < 0.05 was used as the standard for statistical significance. Analyses were calculated with assistance from the Statistical Package for the Social Sciences software (SPSS, version 16.0).

## 3. Results

### 3.1. Behavioral Assessment

**Figure 1:**
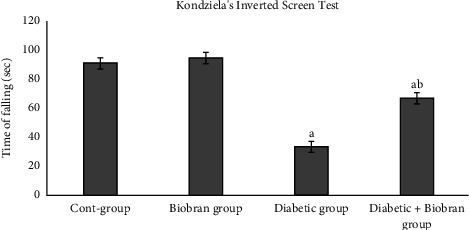
Kondziella's inverted screen test. Values are mean ± *S*.*E*.; each group has *n* = 6. ^a^Significantly different relative to control group, *p* < 0.05. ^b^Significantly different relative to diabetic group, *p* < 0.05.

Following the induction of diabetes, the muscular strength and motor coordination of all rat paws were assessed via Kondziella's inverted screen test (Figure 1). The time of falling decreased significantly (*p* < 0.05) for the untreated diabetic group in comparison with controls. Diabetes affected muscular strength so that rats quickly fell down because they were unable to hold onto the inverted screen. Diabetic rats treated with Biobran had significant increases (*p* < 0.05) in their time of falling in comparison with untreated diabetic rats.

### 3.2. Glucose, Insulin, and Lipid Profile Levels

Serum measurements indicated that there were significantly increased glucose levels (*p* < 0.05) and significantly decreased insulin levels (*p* < 0.05) for the untreated diabetic group relative to control (Table 1). Biobran significantly reversed these effects (*p* < 0.05) for diabetic rats' glucose and insulin levels in comparison with the untreated diabetic rats. The serum measurements also showed significantly increased TC and TG concentrations (*p* < 0.05) for diabetic rats in comparison with control, but Biobran again significantly reversed the effect on serum TC and TG concentrations (*p* < 0.05) relative to the untreated diabetic group.

### 3.3. Hippocampal Oxidative Stress Markers and Antioxidant Enzymes

Brain levels of oxidative stress markers TBARS and NO showed significant elevation (*p* < 0.05) for untreated diabetic rats in comparison with control (Table 2), along with significant reduction (*p* < 0.05) for reduced glutathione (GSH) and the antioxidant enzyme activities of GPX and SOD. Biobran administration was effective at reversing all of these effects. Biobran treatment significantly reduced (*p* < 0.05) elevated TBARS and NO levels and significantly boosted (*p* < 0.05) concentrations of GSH, GPX, and SOD relative to the untreated diabetic group.

### 3.4. Neurochemical Estimations

As indicated in Table 3, the untreated diabetic group displayed significant decreases in brain levels of acetylcholine (ACh), serotonin, and dopamine relative to control. However, oral administration of Biobran significantly (*p* < 0.05) augmented brain levels of ACh, serotonin, and dopamine relative to untreated diabetic rats. In addition, activities of brain AChE and MAO significantly increased (*p* < 0.05) for the untreated diabetic group in comparison with control, while Biobran supplementation to diabetic rats led to significant decreases (*p* < 0.05) in the activities of brain AChE and MAO compared to untreated diabetic group.

### 3.5. Hippocampus Levels of Insulin Receptor (IR), Akt, Inflammatory Cytokines, and Amyloid *β*_1-42_

Brain IR and Akt concentrations had significant decreases (*p* < 0.05) for untreated diabetic rats relative to control, as well as significantly increased (*p* < 0.05) TNF-*α*, NF-*κ*B, IL-1*β*, and amyloid *β*_1-42_ concentrations (Table 4). In contrast, treatment with Biobran to diabetic rats caused significantly increased (*p* < 0.05) concentrations for IR and Akt along with significant decreases (*p* < 0.05) in concentration for TNF-*α*, NF-*κ*B, IL-1*β*, and amyloid *β*_1-42_ relative to untreated diabetic rats.

### 3.6. Histological and Immunohistochemical Assessments

**Figure 2:**
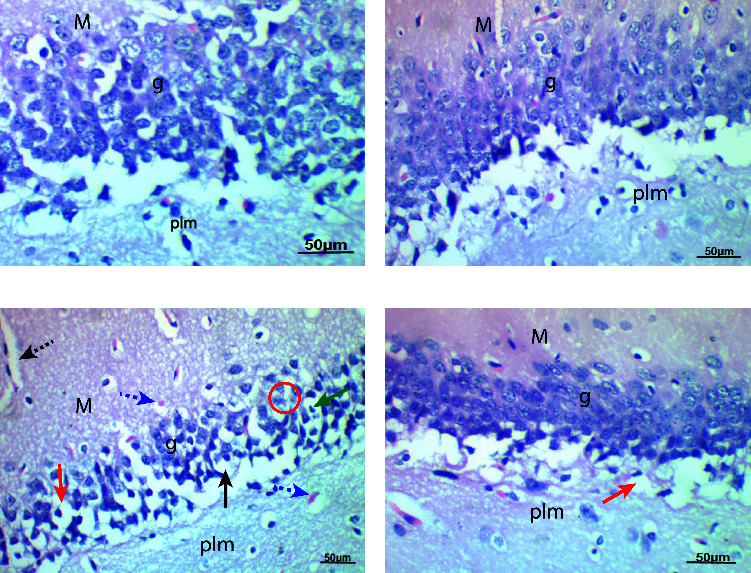
Photomicrographs of sections in the hippocampus of male rats. (a, b) control and Biobran-treated rats show normal polymorphic (plm), normal granular (g), and normal molecular (M) layers. (c) Section from diabetic rat showing degenerated neurons (red circle) with darkly stained pyknotic nuclei (green arrow) enveloped by large perinuclear spaces (black arrow), dilated blood vessels (black dotted arrow) in the molecular layer, and red neurons in polymorphic and granular layers (blue dotted arrow). (d) Diabetic + Biobran-treated rats show marked improvements with few cells with darkly stained nuclei and perinuclear space (red arrow) (H&E, X400).

For control and Biobran-treated rats, histological examination of H&E-stained sections revealed normal hippocampal structure. Untreated diabetic rats showed markedly degenerated neurons with darkly stained pyknotic nuclei enveloped by large perinuclear spaces, dilated blood vessels in the molecular layer, and red neurons in polymorphic and granular layers. Treatment with Biobran to diabetic rats caused improvement with few cells present with darkly stained nuclei and perinuclear space (Figure 2).

**Figure 3:**
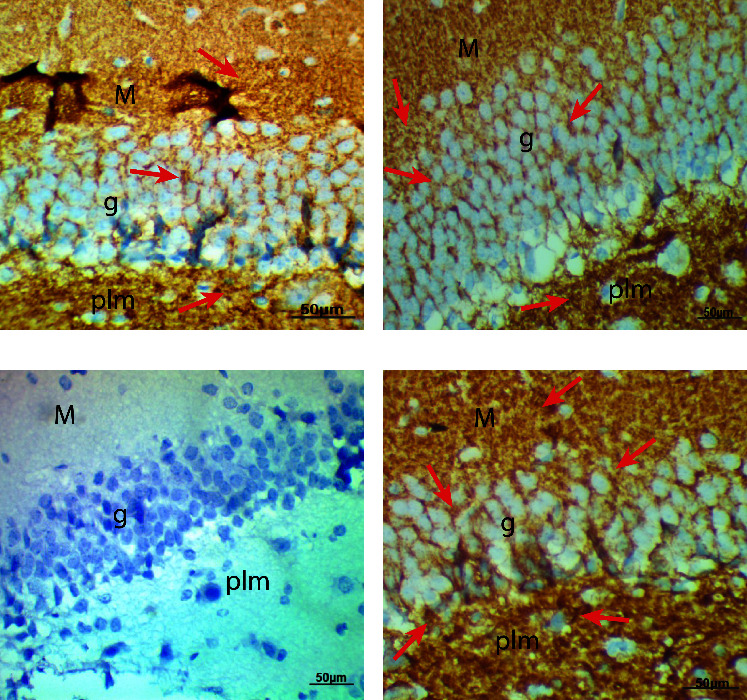
Photomicrographs of rat hippocampi sections revealing immunoreactivity for synaptophysin (CYV). Control (a) and Biobran-treated rat (b) sections show positive reaction for CYV (red arrows). The hippocampus section from diabetic rats (c) reveals weak reaction for CYV. The section from diabetic + Biobran group (d) shows positive reaction for CYV (red arrows) (CYV, X400).

**Figure 4:**
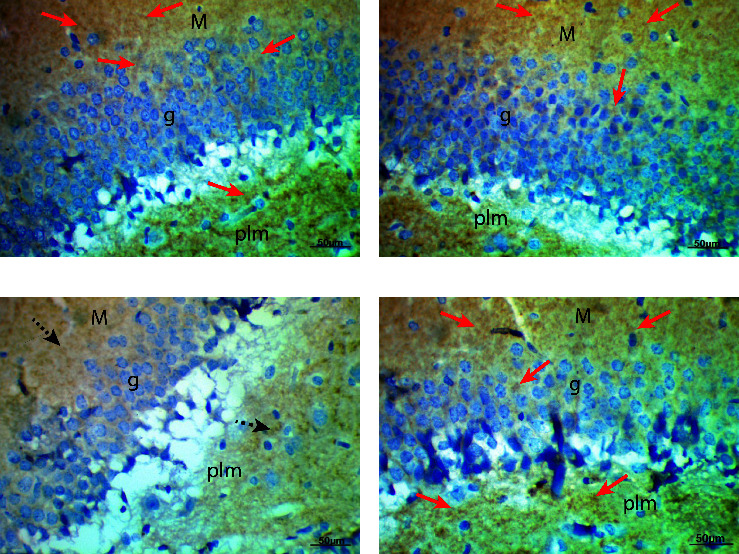
Photomicrographs of rat hippocampi sections revealing immunoreactivity for Ki67. Control (a) and Biobran-treated rat (b) sections showed positive reaction for Ki67 (red arrows). The hippocampus section from diabetic rats (c) revealed weak reaction for Ki67. The diabetic + Biobran group rats (d) showed positive reaction for Ki67 (red arrows) (Ki67, X400).

Synaptophysin, the major synaptic vesicle protein, and the cell proliferation marker Ki67 were used to measure the number of proliferating cells. Control and Biobran-treated rats reacted positively for both synaptophysin and Ki67, while there was a decline in reaction for synaptophysin and Ki67, respectively, in the diabetic group compared to control. Biobran treatment to diabetic rats resulted in significantly increased positive reactions for synaptophysin and Ki67 relative to the untreated diabetic group (Table 5, Figures 3 and 4).

## 4. Discussion

Streptozocin (STZ) treatment was used here to induce T2DM in rats, as manifested in the serum by significantly decreased insulin and significantly increased glucose. The protective effect of the arabinoxylan rice bran product Biobran/MGN-3 against this STZ-induced diabetes and attendant brain damage in rats was then assessed. Biobran is a nutraceutical with health-promoting properties that include potent antioxidant, antiangiogenic, anti-inflammatory, and immunomodulatory properties [9–12, 22, 23]. Supplementation with Biobran to diabetic rats protected against elevations in the levels of glucose, total cholesterol, triglycerides, oxidative stress markers, TBARS, NO, AChE, and MAO, and it led to significant decreases in inflammatory cytokine levels for TNF-*α*, NF-*κ*B, IL-1*β*, and amyloid *β*_1-42_. Furthermore, Biobran ameliorated hippocampal histological alterations and helped restore neuromuscular strength.

Biobran supplementation has been shown to improve blood glucose spikes as well as metabolism of proteins and lipids in diabetic rats [13, 14, 23]. Moreover, recent work has demonstrated that rice bran is effective at lowering blood glucose and improving insulin resistance because it results in increased levels of adiponectin which is linked to whole-body insulin sensitivity [24]. Rice bran diet has the ability to reduce total blood cholesterol, mainly by increasing fecal lipid excretion and regulating lipogenic enzyme activities [24]. Rats fed with Biobran have shown significantly lower triglycerides, total cholesterol, and total protein [13], and Biobran supplementation of diabetic rats has led to significantly lower glucose levels as well as lower total cholesterol levels in comparisons with control rats [14].

The increased blood glucose levels found in diabetes mellitus cause oxidative stress. Several studies showed that STZ-induced diabetes is associated with significantly changed oxidative stress biomarkers [2, 25]. These findings are consistent with the present study, where diabetes was shown to significantly change oxidative stress biomarkers in the hippocampus as indicated by decreased GSH levels and antioxidant enzymes activities of SOD and GPx and by increases in the level of TBARS and NO. However, it is very interesting that Biobran supplementation for STZ-induced diabetic rats caused restorative effects on the functioning of the antioxidant defense system and beneficial decreasing of oxidative stress biomarkers, TBARS, and NO. The antioxidant potential of Biobran noted in this study post i.p. injection of STZ in diabetic rats was further confirmed in our recent model using intracerebroventricular injection of STZ in mice [10]. Those Biobran-fed mice were found to be protected against sporadic Alzheimer's disease and showed significantly increased GSH content and significantly decreased MDA and IL-6 [10]. Reduced inflammatory response indicators and oxidative stress levels have also been found in the study of Biobran's positive effects on mice for alleviating intestinal barrier dysfunction induced by radiation [26].

Neurotransmitters are essential to the health of the mind and body. Low levels for any kind of neurotransmitter can lead to disease development. The current study examines the levels of several important neurotransmitters in rats with STZ-induced diabetes, and they had lower levels of ACh, serotonin, and dopamine as compared to control, while hippocampal activities of AChE and MAO were increased compared to the control, in agreement with previous reports [27, 28]. Biobran treatment of diabetic animals led to significantly elevated (*p* < 0.05) monoamine neurotransmitter levels relative to the diabetic group while decreasing hippocampal AChE and MAO activities.

We noted that the brains of STZ-treated rats have reduced concentrations of IR and Akt and increased concentrations of IL-1*β*, NF-*κ*B, TNF-*α*, and amyloid *β*_1-42_. In diabetic rat studies, STZ has been reported to disrupt hypothalamic signaling either down-stream (PKB) or upstream (IRS-2) of PI3K and/or to block signal transduction through Akt [29]. Our results show that Biobran can enhance the insulin signaling pathway through activation of IR and Akt. Biobran acted as an immunomodulator by reducing the brain's levels of IL-1*β*, NF-*κ*B, TNF-*α*, and amyloid *β*_1-42_. This agrees with our recent study which showed that Biobran supplementation led to significantly declined levels of proinflammatory cytokines and amyloid *β*_1-42_ in mice brains with sporadic Alzheimer's disease [10]. Amyloid plaque's major component, amyloid *β*, exists predominantly as the *Aβ*_1-42_ form, a form that is more likely than *Aβ*_1-40_ to aggregate because it is less soluble. *Aβ*_1-42_ has become a pathological biomarker for neuro-degenerative diseases, and its increased concentration in diabetes strongly suggests it is more likely associated with hyperglycemia [30]. Moreover, rice bran exhibits anti-inflammatory activity, as evidenced by reductions of TNF-*α* and IL-6 (proinflammatory cytokines) and increased IL-10 (anti-inflammatory cytokine) in Raw264.7 macrophage cells [31].

It has been appreciated for a long time that hippocampal formation is responsible for memory and learning. Diseases like diabetes could affect the groups of efferent systems involved with these processes, including sensory processing and integration, affective and social learning, and episodic memory [32]. In the current study, we carried out histological and immunohistochemical analyses of diabetic rats' hippocampi in the presence and absence of Biobran treatment. In H&E-stained hippocampal sections of untreated diabetic rats, there were significant increases in degenerated neurons that had stained pyknotic nuclei enveloped by large perinuclear spaces. In the polymorphic and granular layers, there were red neurons, and in the molecular layer, there were dilated blood vessels. In contrast, histological examination showed Biobran's protective effects. Diabetic rats treated with Biobran showed marked improvement in the abovementioned symptoms. We noted only few cells with darkly stained nuclei and perinuclear space. This could be explained by the ability of Biobran to improve the levels of blood glucose and augment the function of the antioxidant defense system [13, 14, 23].

Further study also showed that diabetes induced a significant decrease of synaptophysin and Ki67-positive cells. Synaptophysin is a major synaptic vesicle protein and is known as a marker of synapses that is affected by several neurodegenerative diseases; several studies have confirmed that diabetes results in the degradation of synaptophysin protein [33, 34]. Spine plasticity and synaptic integrity are key to learning and memory. Diabetes and the resulting insulin signaling disruption have been found to potentiate or exaggerate synaptic damage linked to learning deficits [33]. Earlier studies also reported that hyperglycemia impaired neurogenesis via reduced neuronal proliferation, migration, and maturation and caused cognitive impairments [35]. Our findings show that Biobran can antagonize the suppression of neurogenesis induced by diabetes, indicating that oxidative stress may reasonably be assumed to be indirectly or directly implicated in suppressing neurogenesis in diabetes. Biobran also enhances health-related quality of life for healthy old adults [36]. Our previous results showed that Biobran supplementation of aged subjects caused enhancement in physical and mental component levels in comparison with placebo-treated subjects and baseline values. Taken together, our findings indicate that Biobran is a potent psychoneuroimmune modulatory agent [36].

The neuromuscular impairment caused by diabetic neuropathy is also a well-established phenomenon in type 2 diabetic patients [37, 38] and rodents [39, 40]. In our study, we noted that STZ-induced diabetic rats showed a decline in neuromuscular strength as compared with normal rats as shown by Kondziella's inverted screen test. The neuromuscular impairment caused by diabetic neuropathy is consistent with the results by others [39–41]. Treatment with Biobran helped to restore the neuromuscular strength as demonstrated in increased skeletal muscle strength for Biobran-treated diabetic rats.

## 5. Conclusions

Biobran/MGN-3 was found to have curative effects against STZ-induced cognitive impairment, neurotoxicity, neuroinflammation, and neuronal death. All neurotoxic and oxidative parameters induced by STZ were attenuated. Biobran/MGN-3 improved the levels of the neurochemical transmitters ACh, dopamine, serotonin, AChE, and MAO in the hippocampal tissue. Thus, Biobran administration to diabetic rats attenuates cognitive impairment and synaptotoxicity in the hippocampus by acting on oxidative stress and the IR/Akt/NF-*κ*B pathway. Biobran/MGN-3 could potentially act as a novel therapeutic agent for treating neuro-degenerative disorders.

## Figures and Tables

**Table 1:** Serum levels of glucose, insulin, and lipid profile.

Parameters	Experimental groups			
	Control group	Biobran group	Diabetic group	Diabetic + Biobran group
Glucose (mg/dl)	78.58 ± 2.37	77.53 ± 3.06^b^	454.28 ± 3.24^a^	134.30 ± 1.99^ab^
Insulin (mIU/L)	2.14 ± 0.06	2.31 ± 0.04^ab^	0.72 ± 0.07^a^	1.21 ± 0.03^ab^
Cholesterol (mg/dl)	61.37 ± 1.35	65.10 ± 2.67^b^	186.33 ± 3.01^a^	92.57 ± 3.47^ab^
Triglyceride (mg/dl)	77.05 ± 1.57	77.95 ± 2.05^b^	239.12 ± 4.77^a^	106.03 ± 2.56^ab^

Values are mean ± *S*.*E*.; each group has *n* = 6. ^a^Significantly different relative to control group, *p* < 0.05. ^b^Significantly different relative to diabetic group, *p* < 0.05.

**Table 2:** Levels of brain oxidative stress markers and antioxidant enzymes.

Parameters	Experimental groups			
	Control group	Biobran group	Diabetic group	Diabetic + Biobran group
TBARS (nmol/g tissue)	4.58 ± 0.55	4.85 ± 0.71^b^	21.73 ± 1.73^a^	10.13 ± 1.01^ab^
NO (*μ*mol/g tissue)	5.75 ± 0.42	5.50 ± 0.51^b^	12.10 ± 0.61^a^	8.57 ± 0.53^ab^
GSH (nmol/g tissue)	61.20 ± 1.94	66.82 ± 1.75^b^	22.40 ± 1.50^a^	50.75 ± 3.36^ab^
SOD (U/g tissue)	30.83 ± 1.66	31.00 ± 1.29^b^	15.83 ± 1.78^a^	25.17 ± 1.97^ab^
GPX (U/g tissue)	8.85 ± 0.28	9.40 ± 0.51^b^	5.47 ± 0.37^a^	7.70 ± 0.22^ab^

Values are mean ± *S*.*E*.; each group has *n* = 6. ^a^Significantly different relative to the control group, *p* < 0.05. ^b^Significantly different relative to the diabetic group, *p* < 0.05.

**Table 3:** Neurochemical estimations.

Parameters	Experimental groups			
	Control group	Biobran group	Diabetic group	Diabetic + Biobran group
Ach (U/g tissue)	12.20 ± 0.58	12.48 ± 0.55^b^	5.92 ± 0.70^a^	9.10 ± 0.44^ab^
Dopamine (ng/g tissue)	18.25 ± 0.374	17.92 ± 0.52^b^	5.88 ± 0.32^a^	12.32 ± 0.26^ab^
Serotonin (ng/g tissue)	6.70 ± 0.22	6.62 ± 0.26^b^	2.80 ± 0.29^a^	5.18 ± 0.35^ab^
AchE (pg/g tissue)	9.55 ± 0.52	9.68 ± 0.48 ^ab^	16.20 ± 1.08^a^	11.58 ± 0.26^ab^
MAO (Mu/g tissue)	13.18 ± 0.36	13.53 ± 0.43^b^	23.75 ± 0.75^a^	16.42 ± 0.64^ab^

Values are mean ± *S*.*E*.; each group has *n* = 6. ^a^Significantly different relative to the control group, *p* < 0.05. ^b^Significantly different relative to the diabetic group, *p* < 0.05.

**Table 4:** Brain levels of insulin receptor (IR), Akt, inflammatory cytokines, and amyloid *β*_1-42_.

Parameters	Experimental groups			
	Control group	Biobran group	Diabetic group	Diabetic + Biobran group
IR (ng/g tissue)	13.10 ± 0.24	12.63 ± 0.55^b^	4.90 ± 0.41^a^	8.30 ± 0.45^ab^
Akt (*μ*g/g tissue)	3.87 ± 0.02	4.08 ± 0.04^ab^	0.58 ± 0.01^a^	2.68 ± 0.02^ab^
TNF-*α* (pg/g tissue)	17.02 ± 0.74	16.93 ± 0.80^b^	58.82 ± 2.52^a^	23.65 ± 1.13^ab^
NF-*κ*B (ng/g tissue)	4.35 ± 0.36	4.37 ± 0.54^b^	14.50 ± 1.09^a^	8.73 ± 0.92^ab^
IL-1*β* (pg/g.tissue)	23.28 ± 0.97	19.15 ± 0.69^ab^	37.85 ± 1.71^a^	26.68 ± 1.01^b^
Amyloid *β*_1-42_ (pg/g tissue)	58.08 ± 2.20	54.60 ± 2.00^b^	83.75 ± 1.74^a^	66.18 ± 2.17^ab^

Values are mean ± *S*.E.; each group has *n* = 6. ^a^Significantly different relative to the control group, *p* < 0.05. ^b^Significantly different relative to the diabetic group, *p* < 0.05.

**Table 5:** Changes in hippocampal synaptophysin and Ki67-positive cells (mean number) of male rats in the different experimental groups.

Parameters (unit)	Experimental groups			
	Control group	Biobran group	Diabetic group	Diabetic + Biobran group
Synaptophysin	14.83 ± 0.65	15.67 ± 1.17^b^	2.17 ± 0.48^a^	8.83 ± 0.48^ab^
Ki67	7.67 ± 0.61	8.17 ± 0.40^b^	1.67 ± 0.33^a^	4.80 ± 0.37^ab^

Values are mean ± *S*.*E*.; each group has *n* = 6. ^a^Significantly different relative to the control group, *p* < 0.05. ^b^Significantly different relative to the diabetic group, *p* < 0.05.

## Data Availability

The data that support the findings of this study are available from the corresponding author upon request.
